# The Eaton–Littler Ligament Reconstruction in Thumb Carpometacarpal Joint Instability: Outcomes and Prognostic Factors in 74 Patients

**DOI:** 10.1097/PRS.0000000000011709

**Published:** 2024-09-04

**Authors:** Niek J. Nieuwdorp, Isabel C. Jongen, Caroline A. Hundepool, Mark J. W. van der Oest, Thybout M. Moojen, Ruud W. Selles, J. Michiel Zuidam

**Affiliations:** Rotterdam, the Netherlands; From the Departments of 1 Plastic, Reconstructive, and Hand Surgery; 3 Rehabilitation Medicine, Erasmus Medical Center; 2 Hand and Wrist Center, Xpert Clinics.

## Abstract

**Background:**

The Eaton–Littler ligament reconstruction is widely used for thumb carpometacarpal instability, yet the existing literature lacks a thorough analysis of the outcomes for nontraumatic instability. This study aimed to assess the outcomes of the Eaton–Littler ligament reconstruction and to identify prognostic factors associated with postoperative pain.

**Methods:**

Patients with nontraumatic carpometacarpal joint instability, unresponsive to conservative treatment, were included in this prospective study. The visual analog scale (range 0 to 100) for pain and the Michigan Hand Outcome Questionnaire (MHQ; range 0 to 100) total score were measured at intake and 3 and 12 months postoperatively. Multivariable linear regression was used to analyze the association between preoperative variables and the 12-month MHQ pain score.

**Results:**

Seventy-four patients undergoing Eaton–Littler ligament reconstruction were included. The median visual analog scale pain score improved significantly (*P* < 0.001) from intake (70 [interquartile range, 63 to 78]) to 12 months postoperatively (27 [interquartile range, 7 to 56]). The mean MHQ total score also improved significantly (*P* < 0.001) from intake (52; SD, 13) to 12 months (74; SD, 17). All thumbs were stable at follow-up with preserved range of motion. Grip and pinch strength also improved significantly after surgery. Undergoing a concurrent surgery during ligament reconstruction and a better MHQ pain score at intake were found to be predictors of a favorable postoperative MHQ pain score.

**Conclusions:**

Patient- and clinician-reported outcomes improved significantly at 3 and 12 months after Eaton–Littler ligament reconstruction. The authors advise concurrent hand pathologies resulting from instability (eg, tendinitis, synovitis) to be treated simultaneously during ligament reconstruction.

**CLINICAL QUESTION/LEVEL OF EVIDENCE:**

Therapeutic, III.

The thumb carpometacarpal (CMC) joint provides a wide range of motion (ROM) to the thumb, due to the 2 interlocking saddle shapes opposing each other. The joint capsule and ligaments of the thumb CMC joint play a critical role in ensuring joint stability during movement, as the skeletal congruity provides little stability.^1,2^ Laxity of these stabilizing ligaments results in instability of the CMC joint, manifesting as excessive mobility, pain, functional limitation, and weakness in grip and pinch strength.^2–4^ CMC joint instability may develop from trauma, chronic overuse, generalized ligamentous laxity, or connective tissue disorders (eg, Ehlers–Danlos syndrome).^1–3^ CMC instability is distinguished from CMC osteoarthritis by the absence of degenerative changes in the joint. However, CMC instability is associated with the development of osteoarthritis.^4–6^

The diagnosis of thumb CMC instability primarily relies on clinical presentation and physical examination, in which the stability of the CMC joint is tested by displacing the first metacarpal in a dorsoradial direction and comparing it with the contralateral side. However, in cases of generalized ligamentous laxity, the contralateral side may also exhibit laxity. Radiographic imaging may be used to assess instability and potential degenerative changes.^1,2^

The initial management of CMC instability is nonsurgical, consisting of exercise therapy and orthotics. Conservative treatment often provides successful results, and only 14% of patients convert to surgery over a median follow-up of 2.8 years.^7^ Surgical stabilization is indicated when nonsurgical treatment does not provide sufficient symptom relief.^1–3,7^

Volar ligament reconstruction, first described by Eaton and Littler, is a well-known approach used for CMC joint stabilization. This technique, often referred to as the Eaton–Littler ligament reconstruction, is a nonanatomic ligament reconstruction that supports the anterior oblique ligament, intermetacarpal ligament, and dorsoradial ligament by passing a partial flexor carpi radialis (FCR) tendon graft through a tunnel in the first metacarpal bone.^8^ The Eaton–Littler ligament reconstruction has been reported to demonstrate favorable postoperative outcomes, including restored joint stability, adequate function, and improved pain.^8–13^ However, these results are often based on limited sample sizes using nonstandardized outcome measures, lacking prognostic factors regarding postoperative results. Moreover, current studies often include instability originating from various causes, including both traumatic and nontraumatic causes.^9,11–13^ Trauma-induced cartilage damage, although not always immediately apparent, is believed to affect the success of ligament reconstruction procedures. Therefore, a precise evaluation of the outcomes of the Eaton–Littler ligament reconstruction in patients presenting with nontraumatic CMC instability is of use. Furthermore, knowledge of prognostic factors influencing postoperative pain outcomes might prove to be useful in predicting the postoperative result, providing valuable insights for surgeons and patients in making an informed choice for treatment.

This study aimed to assess the prospectively collected outcomes of the Eaton–Littler procedure in patients presenting with nontraumatic thumb CMC instability. The second aim was to identify prognostic factors associated with postoperative pain.

## PATIENTS AND METHODS

Prospective data were collected as part of routine outcome measurements implemented at our clinic.^14^ Patients presenting with thumb CMC instability treated with the Eaton–Littler ligament reconstruction between December of 2011 and October of 2021 were considered eligible for inclusion. Patients were diagnosed with thumb CMC instability by a certified hand surgeon based on clinical presentation and physical examination, assessing joint instability by dorsoradially displacing the first metacarpal. Imaging was only performed in cases of suspected chondropathy in the CMC or scaphotrapeziotrapezoidal (STT) joint.^8^

Patients were initially treated with exercise therapy and/or an orthosis by a hand therapist. Surgical intervention was considered if nonoperative treatment proved ineffective.^7^

We included patients who completed the visual analog scale (VAS) for pain and the Michigan Hand Outcomes Questionnaire (MHQ) at intake and 3 and 12 months postoperatively. We excluded patients presenting with a history of trauma, chondropathy of the thumb CMC joint (Eaton and Glickel^15^ stage greater than 1) or the STT joint, connective tissue disease, or a history of surgery that could potentially affect the study outcomes. This study adhered to the Strengthening the Reporting of Observational Studies in Epidemiology guidelines.^16^ Ethical approval for our study was granted by the Medical Research Ethical Committee of Erasmus Medical Center. All patients provided written consent for the use of their data.^14^

### Surgical Technique

**Fig. 1.**
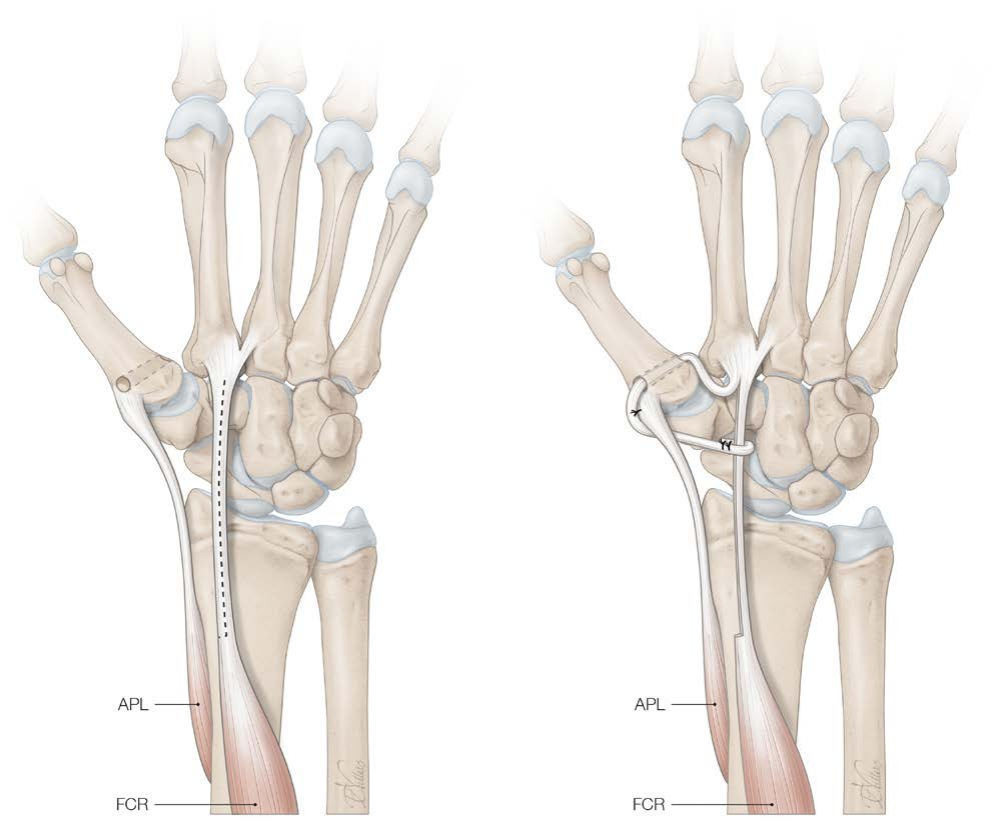
Modified Eaton–Littler ligament reconstruction. (*Left*) A strip of the flexor carpi radialis (*FCR*) tendon is harvested, and a bone tunnel is created through the base of the first metacarpal. (*Right*) The FCR strip is passed through the bone tunnel and sutured upon the abductor pollicis longus (*APL*) tendon and tied to the FCR tendon. (Copyright © 2024 J. Michiel Zuidam, MD, PhD, Esser Masterclass. Published with permission from Dr. Zuidam.)

The ligament reconstruction was performed by 10 surgeons, all certified by the Federation of European Societies for the Surgery of the Hand. All procedures were performed under either general or regional (supraclavicular or axillary) anesthesia. First, a modified Wagner incision was made, and the radial sensory nerve was preserved. The thenar muscles were elevated and a capsulotomy of the trapeziometacarpal joint was performed and the joint was inspected for osteoarthritic changes. The surgeon proceeded with ligament reconstruction if the cartilage was deemed of adequate quality. A synovectomy was performed if considered necessary. Using a drill, a bone tunnel was created from dorsal to volar in the base of the first metacarpal. A transverse skin incision was made on the forearm and a strip of the FCR was harvested using a tendon stripper. The FCR strip was passed through the bone tunnel from volar to dorsal. The strip was then secured to the abductor pollicis longus and the remaining FCR under adequate tension with the thumb placed in palmar abduction, thus performing a minor modification to the original technique^8^ (Fig. 1). The capsule of the thumb CMC joint and the skin were closed, and a plaster was applied with the thumb in radial and palmar abduction.

### Postoperative Rehabilitation

No Kirschner wires were used for immobilization. A standardized postoperative protocol was applied to all patients, consisting of cast splint immobilization for 3 to 5 days, after which the plaster was removed and replaced by a thumb spica splint. Patients did not resume all activities immediately but remained under the supervision of a specialized hand therapist, using a removable brace and receiving intensive hand therapy at least 2 to 3 times a week. Active thumb CMC joint ROM exercises were initiated starting at 5 weeks. At 9 to 13 weeks after surgery, splint use was gradually reduced to restore thumb strength and function.

### Outcome Measures

Primary outcomes included VAS pain scores (0 to 100) and MHQ total scores (0 to 100) at 3 and 12 months postoperatively. We analyzed the number of patients reaching minimal clinically important difference (MCID) for VAS pain score and MHQ total score at 12 months. The MCID values for the VAS pain score and MHQ total score are 23 and 13, respectively.^17^

Secondary outcome measures included MHQ pain and function score, ROM, pinch and grip strength, return to work,^18^ and satisfaction with the treatment result at 12 months.^19^ ROM and strength were measured in a subset of patients as part of routine measurements. These measurements were performed by a qualified hand therapist at the clinic, at intake, and 3 and 12 months postoperatively. Grip strength was measured using an E-LINK Jamar-style dynamometer (Biometrics). Tip, tripod, and key pinch were measured using a dynamometer. Thumb opposition was assessed using the Kapandji score.^20^ Palmar abduction was measured using a caliper to measure the intermetacarpal distance in millimeters. The radial abduction angle was measured in degrees using a goniometer. Return to work was defined as resuming at least half of contracted job duties, based on self-report collected at 6 weeks and 3, 6, and 12 months postoperatively. Because the study was conducted in the Netherlands, all participants received workers’ compensation.^18^

Demographic variables, including age, sex, occupational status, symptom duration, treatment side, and hand dominance, are routinely collected in our database. Moreover, details on the surgical procedure, additional perioperative procedures, and complications were collected through chart review. Complications were classified using the International Consortium for Health Outcomes Measurement Complications in Hand and Wrist Conditions (ICHAW) classification.^21–23^ (**See Table, Supplemental Digital Content 1**, which illustrates the ICHAW tool, http://links.lww.com/PRS/H444.)

### Statistical Analysis

Distribution of the data was evaluated using histograms, quantile–quantile norm plots, and the Shapiro-Wilk test. Normally distributed data were displayed as mean (SD), and skewed data as median (interquartile range [IQR]). For the primary analysis, to compare continuous variables at different time points, a paired *t* test was used for normally distributed data. A Wilcoxon signed-rank test was used for non-normally distributed data. Associations between variables and the 12-month MHQ pain score were analyzed using multivariable linear regression. The variables included in the analysis were selected based on review of the literature and consultations with experts in the field. All assumptions of the linear regression model were checked and satisfied.

We tested for significant differences in demographic characteristics and preoperative scores between included patients (responders) and excluded patients (nonresponders). We also tested for differences in patients with outcomes on ROM and strength (responders) and patients who missed these outcomes (nonresponders). Unpaired *t* test, chi-square test, Fisher exact test, and Wilcoxon rank sum test were used to compare the baseline characteristics. We also performed the Little test to investigate whether data were missing completely at random.^24^

The confidence interval was set at 95% and *P* values less than 0.05 were considered statistically significant. The analyses were performed using R statistical software, version 4.3.0 (R Foundation for Statistical Computing).

## RESULTS

### Demographics and Study Sample

**Fig. 2.**
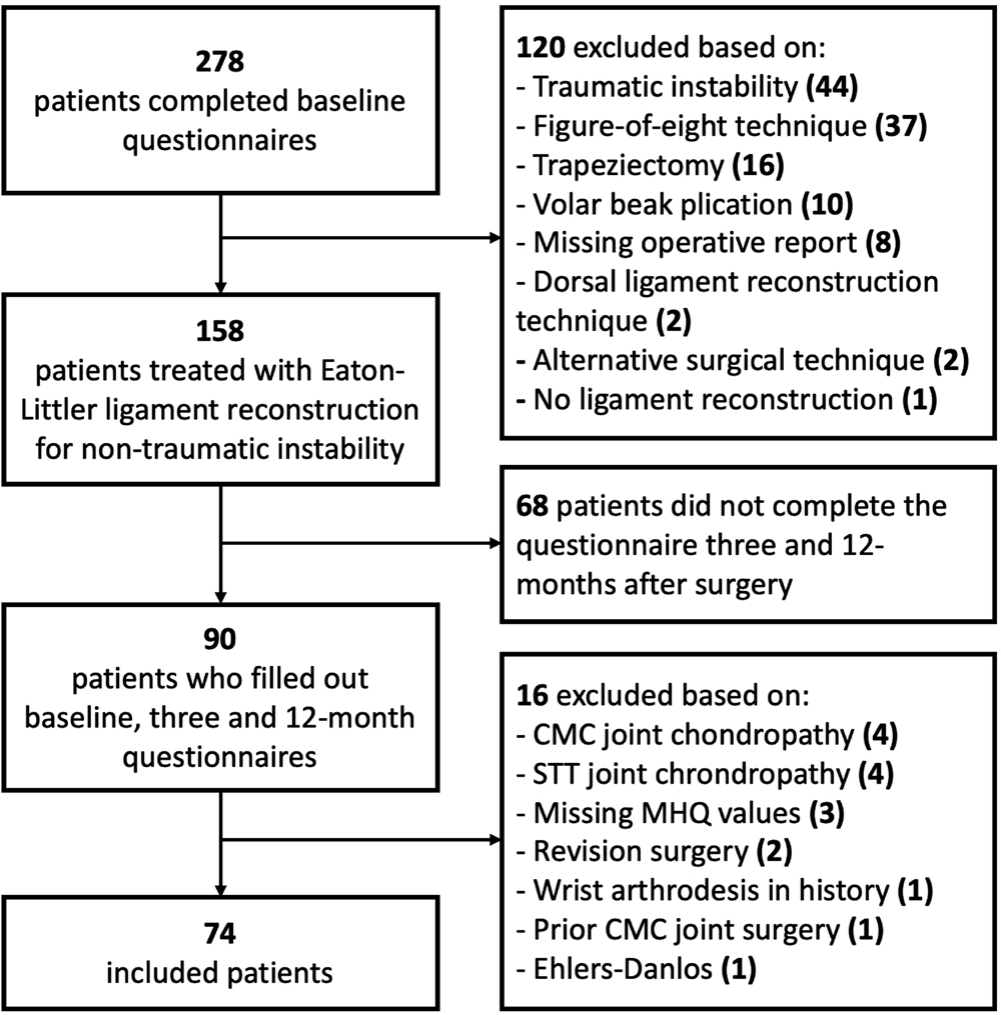
Study flowchart.

Seventy-four patients were included in the final analysis (Fig. 2). The patients were predominantly female (91%), with a median age of 39 years (IQR, 27 to 46 years). The baseline demographic characteristics of the included patients are shown in Table 1. The nonresponder analysis (**see Table, Supplemental Digital Content 2**, which illustrates the nonresponder analysis, http://links.lww.com/PRS/H445) and Little test (*P* = 0.761) suggested that missing data on postoperative VAS pain score and MHQ total score were missing completely at random.

**Table 1.** Characteristics of the Study Sample at Baseline

Characteristics	Value (*n* = 74)
Age, median (IQR), yr	39 (27–46)
Female sex, no. (%)	67 (91)
Symptom duration, median (IQR), mo	18 (9–24)
Dominant side, no. (%)	
Left	10 (14)
Right	64 (87)
Treated side, no. (%)	
Left	31 (42)
Right	43 (58)
Dominant side treated, no. (%)	47 (64)
Occupational intensity, no. (%)	
Unemployed	9 (12)
Light physical labor	26 (35)
Moderate physical labor	29 (39)
Heavy physical labor	10 (14)
Second opinion, no. (%)	15 (20)

### Patient-Reported Pain and Function

**Fig. 3.**
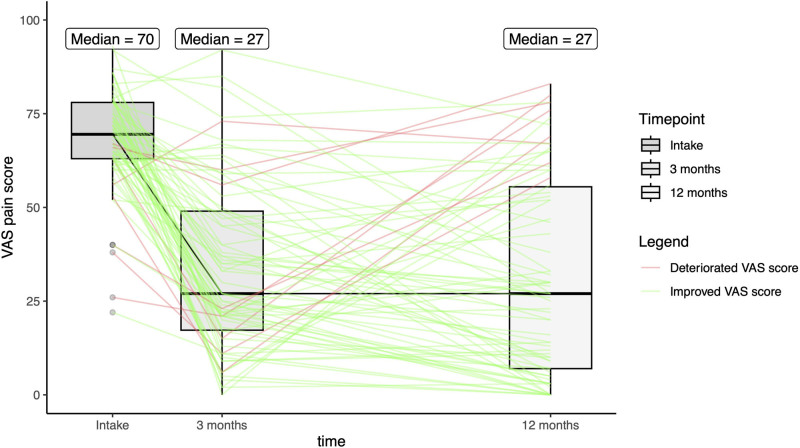
Median VAS pain score before Eaton–Littler ligament reconstruction and 3 and 12 months postoperatively. The *gray boxes* depict the interquartile range. The VAS pain score is plotted for each patient. The *green lines* depict the patients who had improved VAS pain score from intake to 12 months postoperatively. The *red lines* depict the patients who did not have improvement. The *dots* represent outliers. The median VAS pain score improved significantly after Eaton–Littler ligament reconstruction. Large between-subject variability was observed at all time points.

**Fig. 4.**
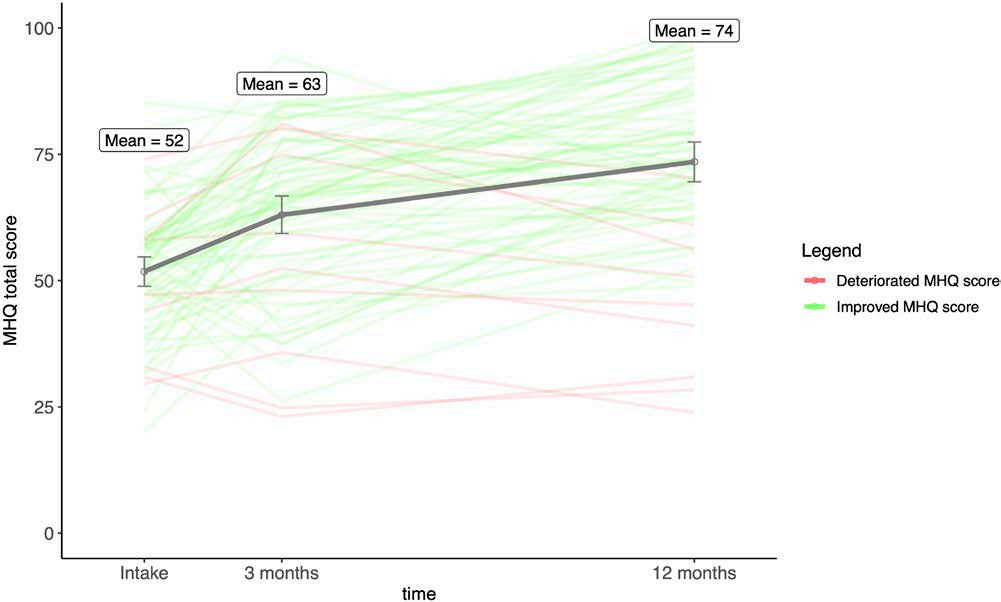
Mean MHQ total score before Eaton–Littler ligament reconstruction and 3 and 12 months postoperatively. The error bars indicate 95% confidence intervals. The MHQ total score is plotted for each patient. The *green lines* depict the patients who improved in MHQ total score from intake to 12 months postoperatively. The *red lines* depict the patients who did not have improvement. The mean MHQ total score improved significantly after Eaton–Littler ligament reconstruction. Large between-subject variability was observed at all time points.

**Fig. 5.**
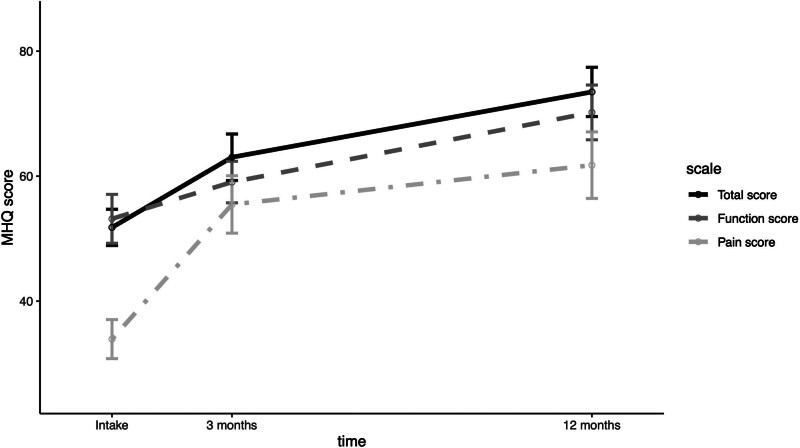
Mean MHQ total, pain, and function scores before Eaton–Littler ligament reconstruction and 3 and 12 months postoperatively. The error bars indicate 95% confidence intervals. Mean MHQ total, pain, and function scores exhibit significant (*P* < 0.001) improvement after Eaton–Littler ligament reconstruction.

Median VAS pain scores improved significantly (*P* < 0.001) from intake (70 [IQR, 6 to 78]) to 12 months postoperatively (27 [IQR, 7 to 56]). Although there was an overall improvement, large between-subject variability was observed at all time points, increasing between the 3- and 12-month postoperative assessments (Fig. 3). The MHQ total score also improved significantly (*P* < 0.001) from intake (52 [SD, 13]) to 3 months (63 [SD, 16]) and 12 months (74 [SD, 17]) (Fig. 4). (**See Table, Supplemental Digital Content 3**, which illustrates the median VAS pain score and mean MHQ total score before and 3 and 12 months after Eaton–Littler ligament reconstruction, http://links.lww.com/PRS/H446.) The mean MHQ pain score and MHQ function subscores also increased significantly (*P* < 0.001) between intake and 12 months postoperatively. The improvement in the MHQ total score is primarily attributed to the substantial improvement in the MHQ pain score, compared with improvement in the MHQ function score (Fig. 5). At 12 months postoperatively, 62% of patients reached the MCID for VAS pain score, and 69% of patients reached the MCID for MHQ total score.

### Range of Motion and Strength

All thumbs were stable based on physical examination at 3 months postoperatively. ROM was preserved, as opposition, palmar abduction, and radial abduction did not differ significantly between intake and 12 months postoperatively (Table 2). At the 12-month assessment, 92% of patients achieved a Kapandji opposition score of 7 or higher. Grip strength, key pinch, and tip pinch all improved significantly between intake and 12 months (Table 3). The Little test (*P* = 0.467) and the nonresponder analysis suggested that data on ROM and strength were missing completely at random. (**See Table, Supplemental Digital Content 4**, which illustrates the nonresponder analysis for ROM and strength measurements, http://links.lww.com/PRS/H447.)

**Table 2.** Active Range of Motion after Eaton–Littler Ligament Reconstruction

Range of Motion	No.	Median (IQR) or Mean ± SD		*P*
		Intake	12 Months	
CMC1 opposition (Kapandji) (0–10)	36	9 (9–10)	9 (9–10)	0.583^a^
CMC1 palmar abduction (IMD), mm	35	64 (58–68)	62 (56–67)	0.227^a^
CMC1 radial abduction angle, degrees	37	50 ± 14	49 ± 8	0.515^b^

CMC1, first carpometacarpal joint; IMD, intermetacarpal distance.

a The *P* value is calculated using the Wilcoxon signed rank test.

b The *P* value is calculated using the paired *t* test.

**Table 3.** Strength Measurement after Eaton–Littler Ligament Reconstruction

Strength Measurement	No.	Median (IQR) or Mean ± SD		*P*
		Intake	12 Months	
Grip strength, kg	40	21.3 (15.9–29.1)	25.1 (20.6–31.6)	<0.001^a^
Key pinch, kg	40	5.0 ± 1.7	6.1 ± 1.8	<0.001^b^
Tip pinch, kg	40	3.0 ± 1.2	3.63 ± 1.2	0.003^b^
Tripod pinch, kg	40	4.1 (3.1–5.3)	4.9 (3.6–5.9)	0.070^a^

a The *P* value is calculated using the Wilcoxon signed rank test.

b The *P* value is calculated using the paired *t* test.

### Satisfaction and Return to Work

At 12 months after surgery, 23% of patients rated their satisfaction with the treatment outcome as excellent, 36% as good, 20% as reasonable, 11% as mediocre, and 9% as poor. A total of 81% of patients reported that they were willing to undergo this treatment again. Return-to-work data were obtained for 61 patients due to 4 missing values and 9 patients without current occupation. During the follow-up, 75% of patients returned to work. The median time to return to work was 12 weeks (95% CI, 10 to 15).

### Complications

ICHAW events were reported in 13 patients (18%). Among the reported events, 3 patients (4%) experienced an ICHAW grade 3B complication, consisting of FCR tendinitis requiring release and a persisting sensory disturbance in the radial nerve. One patient (1%) had an ICHAW grade 2 complication, and 9 patients (12%) experienced an ICHAW grade 1 deviation (Table 4).

**Table 4.** Overview of ICHAW Events during the First Year after Eaton–Littler Ligament Reconstruction^a^

Grade	No. (%) of Patients	Specifications
None	61 (82)	—
1	9 (12)	2 scar adhesions requiring hand therapy, 2 cases of suture inflammation, 3 temporary sensory disturbances in the radial nerve, 1 case of recurrent pain requiring additional hand therapy, 1 case of recurrent pain requiring additional analgesics
2	1 (1)	1 case of persistent synovitis requiring steroid injection
3B	3 (4)	2 case of FCR tendinitis requiring release (6 and 12 months postoperatively),^b^ 1 persisting sensory disturbance in the radial nerve

a Overview of the number of ICHAW events and the number of patients who experienced that event, according to the ICHAW tool,^21–23^ during the first year after Eaton–Littler ligament reconstruction.

b Months between ligament reconstruction and surgery for the complication.

### Concurrent Surgical Procedures

Among the patients included in the study, 15 (20%) underwent an additional surgical intervention during the Eaton–Littler ligament reconstruction. Many of these concurrent procedures were performed to treat pathologies resulting from generalized joint laxity, such as tendinitis, volar plate laxity, and synovitis. The most frequent concurrent procedures included de Quervain release (*n* = 5), carpal tunnel release (*n* = 2), and thumb trigger finger release (*n* = 2). A complete overview of all concurrent surgical procedures is provided in the Supplemental Digital Content. (**See Table, Supplemental Digital Content 5**, which provides an overview of concurrent surgical procedures performed during Eaton–Littler ligament reconstruction, http://links.lww.com/PRS/H448.)

### Prognostic Factors

Our model revealed that 2 variables were significantly associated with a higher 12-month postoperative MHQ pain score, indicating a more favorable outcome. The first variable was undergoing a concurrent surgery during ligament reconstruction (β, 13.93; 95% CI, 0.97 to 26.88). Patients undergoing a concurrent surgery had 14 points higher postoperative MHQ pain score on average, compared with patients not undergoing a concurrent surgery. The second variable was a more favorable MHQ pain score at intake (β, 0.55; 95% CI, 0.13 to 0.96). For each additional MHQ pain score point at baseline, the postoperative MHQ pain score increased, on average, by 0.55 points. The other variables (age, duration of complaint, MHQ function score at intake, and MHQ satisfaction score at intake) were not significantly associated with the postoperative MHQ pain score at 12 months. The R^2^ of our model was 0.16. The outcomes of our multivariable regression model are depicted in Table 5.

**Table 5.** Multivariable Linear Regression Analysis for Postoperative MHQ Pain Score 12 Months after Surgery

Variable	β	95% CI	*P*
Age, yrs	−0.00	−0.51 to 0.51	0.992
Duration of complaint, mo	−0.04	−0.30 to 0.23	0.789
Concurrent surgery	13.93	0.97 to 26.88	0.035^a^
MHQ pain score at intake	0.55	0.13 to 0.96	0.010^a^
MHQ function score at intake	−0.19	−0.59 to 0.21	0.340
MHQ satisfaction score at intake	0.34	−0.09 to 0.76	0.118
Adjusted R^2^		0.16	

a Statistically significant (*P* < 0.05).

## DISCUSSION

This study aimed to assess the patient- and clinician-reported outcomes of 74 patients with nontraumatic thumb CMC instability undergoing Eaton–Littler ligament reconstruction. During a 12-month follow-up period, a significant improvement in pain, function, and grip and pinch strength was observed. All thumbs were stable at follow-up, with preserved ROM. The outcomes presented are in line with the current literature, as previous records of the technique dating back to 1973 have consistently demonstrated good or excellent results in the majority of patients across various follow-up periods.^8,9,11–13^ However, the inclusion of posttraumatic instability, nonstandardized outcome measures, limited sample sizes, and variations in follow-up duration in the literature complicate the assessment of the technique’s viability for nontraumatic instability. Spekreijse et al.^10^ reported on the Eaton–Littler procedure in a cohort of patients with nontraumatic thumb CMC instability. However, their study lacks insights into intersubject variability and predictors of postoperative pain, and contains missing values at 12 months. In contrast, our study, featuring complete data for 74 patients, offers a more accurate insight into the efficacy of the technique.

Whereas our outcomes of the Eaton–Littler ligament reconstruction are satisfactory, substantial intersubject variability in pain and function is observed. To provide insight into factors influencing the outcome and the observed variability, we identified predictors of a favorable 12-month postoperative MHQ pain score. Our analysis showed that undergoing a concurrent surgery during ligament reconstruction is a predictor of a higher (better) postoperative MHQ pain score, adjusting for other variables in the model (eg, MHQ pain score and MHQ function score at intake). We hypothesize that undergoing concomitant surgery during ligament reconstruction contributes to a better postoperative pain score by addressing concurrent hand or wrist pathologies that might otherwise hinder recovery or cause subsequent issues. This is particularly pertinent for patients with generalized ligament laxity, who have an increased susceptibility to tendon-related issues. We therefore advise patients presenting with multiple pathologies of the hand or wrist to be treated simultaneously during ligament reconstruction. Second, our analysis indicated an association between the intake and postoperative MHQ pain scores, thus underscoring the importance of preoperative pain management through conservative treatment.^7^ The R^2^ of our model was 0.16, indicating that other measured and unmeasured factors contribute to the variance in outcomes. Adding additional factors to future analysis might provide valuable insight, as our sample size constrained us from performing a more extensive analysis.

Our analysis indicated a subgroup of patients with remaining pain after ligament reconstruction. We hypothesize that the remaining pain might be attributed to unnoticed cartilage wear, although throughout our study the cartilage was examined and determined to be adequate for stabilization during each surgery. Thorough inspection of the joint surfaces remains of utmost importance during surgery. Another hypothesis explaining the persisting postoperative pain is the potential development of concurrent hand pathology due to generalized laxity during the follow-up, which remained untreated. Further studies are needed to validate these hypotheses and explore alternative treatment options, aiming to optimize outcomes for this patient group.

Our study has limitations. Patients were asked whether there had been a traumatic injury to the thumb preceding the symptoms, potentially introducing recall bias. In addition, we only included patients who filled out baseline, 3-month, and 12-month questionnaires, thereby introducing selection bias into the study. However, we are confident that our findings are generalizable, because our analysis indicated that data were missing completely at random. A strength of our study lies in our strict inclusion and exclusion criteria, enabling the precise reporting of outcomes for patients with exclusively nontraumatic and nonarthritic instability. A second strength of our study lies in the prospective nature of data collection and the substantial sample size, making our results suitable for patient counseling. Our findings of prognostic factors are valuable for surgeons, enabling them to make more accurate predictions of postoperative pain levels.

## CONCLUSIONS

The Eaton–Littler ligament reconstruction demonstrates favorable results, with significant improvements in patient- and clinician-reported outcomes at both 3 and 12 months postoperatively. Our analysis revealed that a better preoperative MHQ pain score and undergoing concurrent surgery during ligament reconstruction predict a better postoperative pain outcome. We therefore advise simultaneously treating concurrent hand pathologies resulting from instability during ligament reconstruction. Our results underscore the importance of preoperative conservative therapy.

## APPENDIX

The collaborators of the Hand-Wrist Study Group are as follows: R. A. M. Blomme, J. M. Smit, K. Harmsen, H. Halbesma, G. M. Vermeulen, J. P. de Schipper, J. H. van Uchelen, O. T. Zöphel, J. S. Souer, L. E. Lopez, A. Fink, R. van Huis, P. A. A. Pennehouat, K. Schoneveld, G. D. Arends, R. Feitz, L. Hoogendam, S. E. R. Hovius, Y. E. van Kooij, J. E. Koopman, M. J. W. van der Oest, W. A. de Ridder, L. Sikking, H. P. Slijper, M. H. P. ter Stege, J. S. Teunissen, R. M. Wouters, N. L. Loos, N. H. A. Mendelaar, L. van Wijk, W. R. Bijlsma, J. W. Colaris, L. S. Duraku, E. P. A. van der Heijden, C. A. Hundepool, and J. M. Zuidam.

## DISCLOSURE

The authors have no financial interests to disclose. The authors received no financial support for and have no potential conflict of interest in the research, authorship, or publication of this article.

## ACKNOWLEDGMENT

The authors thank the patients who completed questionnaires as part of their clinical care and consented to the anonymous use of their data in this study, as well as the members of the Hand-Wrist Study Group and the staff of the Xpert Clinics and Equipe Zorgbedrijven for their contributions to the routine outcome measurements forming the foundation of this article.

## Supplementary Material


