# Supplementary material for: The Eaton–Littler Ligament Reconstruction in Thumb Carpometacarpal Joint Instability: Outcomes and Prognostic Factors in 74 Patients
Source: Plast Reconstr Surg. 2024 Sep 4;155(3):533–42. doi: 10.1097/PRS.0000000000011709 (PMC11845075; doi:10.1097/PRS.0000000000011709)
Supplement: Supplementary file 1 [file prs-155-533e-s001.pdf]

Supplemental Digital Content 1. Table that illustrates the ICHOM Complications in Hand and Wrist conditions (ICHAW) tool.[21-23]

**Grade**	**Definition, to occur within the final time point of the relevant track**
Grade I:	Any deviation from the normal treatment course without the need for surgical, endoscopic and radiological interventions. Acceptable therapeutic regimens are: extra analgesics and additional hand therapy/ splinting/ cast. This grade incudes e.g.: tendinitis, scar tenderness, temporary sensory disturbances, etc.<br>Complex Regional Pain Syndrome is excluded from this grade (see Grade III-C).
Grade II:	Any deviation from the normal treatment course requiring antibiotics, steroid injections or other pharmacological treatment not listed in Grade I. Also included are wound infections and hematoma's not needing anesthesia. Complex Regional Pain Syndrome is excluded from this grade (see Grade III-C).
Grade III:<br><br>A:<br><br>B:<br><br>C:	Any deviation from the normal treatment course requiring surgical, endoscopic or radiological intervention. Also, this includes tendinitis, scar tenderness, persistent pain, etc. not responding to conservative therapy, drugs or injections.<br><br>Minor surgical intervention under local anesthesia (e.g. irritating K wire, suture removal subcutaneously)<br><br>Major surgical intervention under regional or general anesthesia (e.g. repeat surgery, tenolysis, neurolysis, nerve repair or surgery for tendon rupture, breaking of plate, non-union, initial prosthesis failure)<br><br>Complex Regional Pain Syndrome, diagnosed using Budapest* criteria, independent of the initiated treatment
