# Supplementary material for: The Eaton–Littler Ligament Reconstruction in Thumb Carpometacarpal Joint Instability: Outcomes and Prognostic Factors in 74 Patients
Source: Plast Reconstr Surg. 2024 Sep 4;155(3):533–42. doi: 10.1097/PRS.0000000000011709 (PMC11845075; doi:10.1097/PRS.0000000000011709)
Supplement: Supplementary file 2 [file prs-155-533e-s002.pdf]

Supplemental Digital Content 2. Table that illustrates the nonresponder analysis.

Variable	Excluded patients (N=84)	Included patients (N=74)	*P*-value
Age, median [IQR]	36 [29-45]	39 [27-46]	0.900
Sex, female N (%)	75 (89)	67 (91)	1.000
Symptom duration mo., median [IQR]	16 [8-32]	18 [9-24]	0.813
Dominant side, N (%)			0.805
Left	9 (11)	10 (14)	
Right	74 (88)	64 (87)	
Both	1 (1)	0 (0)	
Treated side, N (%)			1.000
Left	35 (42)	31 (42)	
Right	49 (58)	43 (58)	
Dominant side treated, N (%)	46 (55)	47 (64)	0.340
Occupational intensity, N (%)			0.232
Unemployed	20 (24)	9 (12)	
Light physical labor	21 (25)	26 (35)	
Moderate physical labor	32 (38)	29 (39)	
Heavy physical labor	11 (13)	10 (14)	
Preoperative VAS pain score, median [IQR]	75 [63-80]	70 [63-78]	0.139
Preoperative MHQ score, mean (SD)			
Total score	49 (14)	52 (13)	0.248
Pain score	33 (16)	34 (14)	0.660
Function score	49 (15)	53 (17)	0.136

SD standard deviation, *IQR* interquartile range, *N* number of patients, *VAS* Visual Analogue Scale, *MHQ* Michigan Hand Outcomes Questionnaire.
