# Supplementary material for: The Eaton–Littler Ligament Reconstruction in Thumb Carpometacarpal Joint Instability: Outcomes and Prognostic Factors in 74 Patients
Source: Plast Reconstr Surg. 2024 Sep 4;155(3):533–42. doi: 10.1097/PRS.0000000000011709 (PMC11845075; doi:10.1097/PRS.0000000000011709)
Supplement: Supplementary file 3 [file prs-155-533e-s003.pdf]

Supplemental Digital Content 3. Table that illustrates the median VAS pain score and mean MHQ total score before Eaton-Littler ligament reconstruction and three and 12 months postoperative.

	Median [IQR] or Mean (SD)			***P* for change over three months**	***P* for change between three and 12-months**	***P* for change over 12 months**
	**Intake**	**3-months**	**12-months**			
VAS pain score (0 – 100)	70 [63–78]	27 [17–49]	27 [7–56]	<0.001+	0.122+	<0.001+
MHQ total score (0 – 100)	52 (13)	63 (16)	74 (17)	<0.001*	<0.001*	<0.001*

SD standard deviation, *IQR* interquartile range, *VAS* Visual Analogue Scale, *MHQ* Michigan Hand Outcomes Questionnaire.

+ The P-value is calculated using the Wilcoxon signed-rank test.

*The P-value is calculated using the paired t-test.
