# Supplementary material for: The Eaton–Littler Ligament Reconstruction in Thumb Carpometacarpal Joint Instability: Outcomes and Prognostic Factors in 74 Patients
Source: Plast Reconstr Surg. 2024 Sep 4;155(3):533–42. doi: 10.1097/PRS.0000000000011709 (PMC11845075; doi:10.1097/PRS.0000000000011709)
Supplement: Supplementary file 4 [file prs-155-533e-s004.pdf]

Supplemental Digital Content 4. Table that illustrates the nonresponder analysis for range of motion (ROM) and strength measurements.

Variable	Patient without ROM and strength measurements (N=34)	Patient with ROM and strength measurements (N=40)	P-value
Age, median [IQR]	39 [28-42]	38 [27-48]	0.529
Sex, female N (%)	32 (94)	35 (88)	0.568
Symptom duration mo., median [IQR]	12 [8-24]	22 [12-24]	0.202
Dominant side, N (%)			0.745
Left	4 (12)	6 (15)	
Right	30 (88)	34 (85)	
Treated side, N (%)			0.725
Left	13 (38)	18 (45)	
Right	21 (62)	22 (35)	
Dominant side treated, N (%)	21 (62)	26 (65)	0.963
Occupational intensity, N (%)			0.918
Unemployed	5 (15)	4 (10)	
Light physical labor	11 (32)	15 (38)	
Moderate physical labor	13 (38)	16 (40)	
Heavy physical labor	5 (15)	5 (13)	
Preoperative VAS pain score, median [IQR]	68 [63-76]	71 [63-78]	0.918
3-months VAS pain score, median [IQR]	32 [21-58]	23 [17-47]	0.334
Preoperative MHQ score, mean (SD)			
Total score	51 (12)	53 (13)	0.574
Pain score	30 (13)	37 (14)	0.035
Function score	53 (19)	53 (16)	0.915
3-months MHQ score, mean (SD)			
Total score	62 (16)	64 (16)	0.578
Pain score	52 (20)	59 (20)	0.163
Function score	60 (14)	58 (15)	0.667

SD standard deviation, IQR interquartile range, N number of patients, VAS Visual Analogue Scale, MHQ Michigan Hand Outcomes Questionnaire.
