# Supplementary material for: The Eaton–Littler Ligament Reconstruction in Thumb Carpometacarpal Joint Instability: Outcomes and Prognostic Factors in 74 Patients
Source: Plast Reconstr Surg. 2024 Sep 4;155(3):533–42. doi: 10.1097/PRS.0000000000011709 (PMC11845075; doi:10.1097/PRS.0000000000011709)
Supplement: Supplementary file 5 [file prs-155-533e-s005.pdf]

Supplemental Digital Content 5. Table providing an overview of concurrent surgeries during Eaton-Littler ligament reconstruction.

Type of surgery	Number
De Quervain's release	5 (7%)
Carpal tunnel release	2 (3%)
Thumb trigger finger release	2 (3%)
Scaphotrapeziotrapezoid joint ganglion extirpation	1 (1%)
Volar plate reinsertion at the metacarpophalangeal joint of the thumb	1 (1%)
Tenolysis of the second and third ray	1 (1%)
Ganglion extirpation and neurolysis of a branch of the radial nerve at the thumb	1 (1%)
Posterior interosseus nerve neurectomy and synovectomy of the scapholunate joint, and plication of the wrist capsule	1 (1%)
Neurectomy of the posterior interosseus nerve and synovectomy of the scapholunate joint	1 (1%)

Values are reported as the number of cases with a percentage computed over the entire study sample (N = 74).
